# Association of structural connectivity with functional brain network segregation in a middle-aged to elderly population

**DOI:** 10.3389/fnagi.2024.1291162

**Published:** 2024-02-01

**Authors:** Maximilian Schulz, Marvin Petersen, Bastian Cheng, Götz Thomalla

**Affiliations:** Department of Neurology, University Medical Centre Hamburg-Eppendorf, Hamburg, Germany

**Keywords:** age, resting state functional MR imaging, structural connectivity, functional connectivity, functional segregation

## Abstract

**Introduction:**

The deterioration of white matter pathways is one of the hallmarks of the ageing brain. In theory, this decrease in structural integrity leads to disconnection between regions of brain networks and thus to altered functional connectivity and a decrease in cognitive abilities. However, in many studies, associations between structural and functional connectivity are rather weak or not observed at all. System segregation, defined as the extent of partitioning between different resting state networks has increasingly gained attention in recent years as a new metric for functional changes in the aging brain. Yet there is a shortage of previous reports describing the association of structural integrity and functional segregation.

**Methods:**

Therefore, we used a large a large sample of 2,657 participants from the Hamburg City Health Study, a prospective population-based study including participants aged 46–78 years from the metropolitan region Hamburg, Germany. We reconstructed structural and functional connectomes to analyze whether there is an association between age-related differences in structural connectivity and functional segregation, and whether this association is stronger than between structural connectivity and functional connectivity. In a second step, we investigated the relationship between functional segregation and executive cognitive function and tested whether this association is stronger than that between functional connectivity and executive cognitive function.

**Results:**

We found a significant age-independent association between decreasing structural connectivity and decreasing functional segregation across the brain. In addition, decreasing functional segregation showed an association with decreasing executive cognitive function. On the contrary, no such association was observed between functional connectivity and structural connectivity or executive function.

**Discussion:**

These results indicate that the segregation metric is a more sensitive biomarker of cognitive ageing than functional connectivity at the global level and offers a unique and more complementary network-based explanation.

## Introduction

Advanced age is linked to a variety of changes in brain structure and neurobiology, as well as alterations in functional brain activity, which occur throughout the human lifespan (Raz et al., 2005; Goh and Park, 2009; Park and Reuter-Lorenz, 2009). Even in the absence of obvious disease, this development is associated with cognitive decline that negates the benefits of increased life expectancy in modern society (Lindenberger and Baltes, 1994; PSYCNET, 2022). Cognitive abilities depend on the efficient functioning of distributed brain networks connected by white matter pathways (Lawrence et al., 2014). Deterioration of these white matter structures due to reduced blood flow, decline in neurotrophic factors and loss of neurons and myelin (Wassenaar et al., 2019; Kong et al., 2020; Hoagey et al., 2021) leads to subtle anatomical disconnection between brain regions that normally function together (O’Sullivan et al., 2001), and in that way to alterations in the functional properties of coordinated brain networks (Salat et al., 2005; Andrews-Hanna et al., 2007). Indeed, there is indication for a relationship between compromised white matter integrity and impaired functional connectivity, also referred to as “disconnection syndrome” (O’Sullivan et al., 2001). Several studies report a positive association between regional white matter integrity and functional connectivity in older adults (Andrews-Hanna et al., 2007; Chen et al., 2009).

However, other studies examining the association between functional connectivity and white matter measurements found only weak or no links between functional and structural connectivity (Fjell et al., 2017; Tsang et al., 2017). Regardless of the common neurobiological basis, the relationship between structural and functional connectivity appears to be complex, and regions with few or no direct structural connections can show high functional connectivity (Damoiseaux and Greicius, 2009; Horn et al., 2014). Furthermore, the cross-sectional patterns of structural and functional connectivity varies across different age groups, mirroring the age-related patterns observed in white and gray matter, which serve as the basis for measuring structural and functional connectivity (Walhovd et al., 2011), and a longitudinal study showed an independent change of functional and structural connectivity over time (Fjell et al., 2017). While the decrease in structural connectivity with increasing age is consistent across the literature (Madden et al., 2020), functional connectivity between cortical regions on the other hand reveals increases and decreases with age in different regions of the brain (Biswal et al., 2010; Tomasi and Volkow, 2012; Betzel et al., 2014; Song et al., 2014). These differences in reported age trajectories and the rich literature supporting the idea of an imperfect match between structure and function necessitate the investigation of new parameters that go beyond a simple one-to-one mapping of structural and functional connectivity.

The human brain is organized in a modular functional network architecture (Ferrarini et al., 2009; He et al., 2009) characterized by segregated brain networks with dense connections within and sparse connections between these networks (Bullmore and Sporns, 2012). This brain organization is thought to benefit specialized or segregated information processing (Bullmore and Sporns, 2012) by staying positively connected to regions supporting similar functions and negatively to those not strongly involved in a specific task (Antonenko and Flöel, 2014). In line with these observations previous studies have found a positive correlation between cognitive processes and the degree of network segregation at different levels of organization (Chan et al., 2014; Gu et al., 2015; Cohen and D’Esposito, 2016; Grady et al., 2016). However, the process of ageing is associated with a progressive degeneration of this functional network architecture (Sala-Llonch et al., 2015; Damoiseaux, 2017; Wig, 2017), and functional networks become less distinct in old adults, quantified as decreased functional network segregation (Chan et al., 2014; Geerligs et al., 2015). This pattern is more pronounced in individuals over 50 years of age (Chong et al., 2019) with older adults exhibiting lower category-specific activation in brain regions sensitive to specific stimuli (Park et al., 2004) and additional activation of other brain areas (Cabeza, 2002; Logan et al., 2002; Gutchess et al., 2005; Morcom et al., 2007).

Although reduced segregation is increasingly defined as a hallmark of ageing in the light of these outcomes, there is a paucity of previous reports describing the association of structural integrity and functional segregation. While one longitudinal study was able to attribute the decline in functional segregation in part to the change in white matter integrity (Pedersen et al., 2021) and another paper found a link between cerebrovascular elasticity, increased cortical atrophy and white matter abnormalities with decreased network segregation (Kong et al., 2020). Madden et al. (2020) reported no direct influence of structural network properties on the age-related decline in functional system separation.

Based on the idea that age-related disruptions in the structural wiring of the brain potentially disturb the balance of connectivity within and between networks, with functional connections between structurally disconnected regions relying on more indirect pathways involving different networks. We presume that the decline in segregation is a more reliable functional pattern that better represents age-related changes in brain activity than functional connectivity. We used a large sample of 2,657 participants, to investigate in an exploratory attempt whether there is a relationship between age-related differences in structural connectivity and functional segregation and whether this relationship is stronger than between structural connectivity and three measures of functional connectivity. We hypothesized first that there is at least a partial effect of structural connectivity on functional segregation. Second we hypothesized that functional segregation, which accounts for the ratio within- and between-network connectivity is a more sensitive biomarker for functional variability in the process of aging and therefore relates stronger to structural differences than functional connectivity.

Moreover, we analyzed in a second step the relationship between functional segregation and executive cognitive function and tested whether this relationship is stronger than that between functional connectivity and executive cognitive function. Since this cohort was relatively healthy with a limited age range between 45 and 74 years, executive function was used as the only cognitive measure, as it appears to be the most susceptible to age-related decline among the various cognitive measures (Wecker et al., 2000; Verhaeghen and Cerella, 2002; Madden et al., 2017). We hypothesized that functional segregation is associated more strongly with cognitive variation than functional connectivity.

## Materials and methods

### Study population

Here, we investigated cross-sectional clinical and imaging data from a subgroup of the first 10,000 participants from the Hamburg City Health Study (HCHS). As described previously the HCHS is an ongoing, single-center, prospective cohort study examining randomly selected citizens of the city of Hamburg, Germany, aged 45 to 74 years at time of invitation (Jagodzinski et al., 2020). Participants were enrolled between 2016 and 2018 and underwent an in-depth multi-organ baseline examination with emphasis on imaging to identify risk factors, prevalence, and prognostic factors for major chronic diseases. All baseline evaluations included standardized neuropsychological examinations by specifically trained medical professionals, while brain MRI was conducted in a subgroup of 2,657 participants. We studied subjects for whom complete data were available, resulting in a cohort of 2,245 participants. None of the participants were screened for MCI or dementia at the start of the study or excluded from a test due to their cognitive performance.

### MRI acquisition

Images were acquired using a 3-T Siemens Skyra MRI scanner (Siemens, Erlangen, Germany). Acquisitions were performed with a protocol as described in previous work (Jagodzinski et al., 2020; Petersen et al., 2020). In detail, for 3D T1-weighted anatomical images, rapid acquisition gradient-echo sequence (MPRAGE) was used with the following sequence parameters: TR = 2500 ms, TE = 2.12 ms, 256 axial slices, ST = 0.94 mm, and IPR = 0.83 mm × 0.83 mm. Resting-state functional images were measured with the following sequence parameters: TR = 3000 ms; TE = 32 ms; flip angle = 90 degrees; FOV = 192 mm × 192 mm; matrix = 64 × 64; slices = 46; slice thickness = 3 mm; slice gap = 0 mm; effective voxel resolution = 3.0 mm × 3.0 mm × 3.0 mm. For single shell diffusion-weighted imaging (DWI), 75 axial slices were obtained covering the whole brain with gradients (b = 1000 s/mm^2^) applied along 64 non-collinear directions with the following sequence parameters: repetition time (TR) = 8500 ms, echo time (TE) = 75 ms, slice thickness (ST) = 2 mm, in-plane resolution (IPR) = 2 mm × 2 mm, anterior–posterior phase-encoding direction, 1 B0 volume.

### Functional and structural connectomes

Structural connectomes were reconstructed employing QSIprep (version 0.14.2) (Cieslak, 2015). Subject representative structural connectomes were obtained via distance-dependent consensus thresholding (Betzel et al., 2019). A method in which the selection of edges in the structural connectome uses a consensus-based threshold that depends on the distance between brain regions instead of a fixed threshold for the connections, so that the connections are selected not only based on their strength, but also depending on the spatial distance between the brain regions involved. Upon preprocessing of resting-state functional MRI data with fMRIPrep (version 20.2.6), HCHS functional connectomes were computed with xcpEngine (version 1.2.3) with denoising based on global signal regression (GSR) and ICA-AROMA (Pruim et al., 2015; Ciric et al., 2017; Esteban et al., 2019). HCHS structural and functional data were parcellated according to the Schaefer-atlas, dividing the brain into 200 contiguous and uniform regions of interest (ROIs) (Schaefer et al., 2018). In addition, the Schaefer Atlas enables the assessment of seven intrinsic functional networks at the macro scale. Of these seven networks, five associative resting state networks were extracted, which contained the default mode network, dorsal attention network, salience network, cognitive control network and the limbic system. All regions of the resting state networks were determined *a priori* by the Schaefer atlas and were thus unique in each resting state network without overlapping between networks. In functional connectomes, the individual mean time series were extracted for each region of interest (ROI). Finally, values of the Pearson’s correlation coefficients were calculated between each pair of ROIs for each subject, which resulted in a square undirected correlation matrix representing the whole cortex and each resting state network. Due to erroneous negative associations possibly induced by the GSR, negative correlations within functional connectomes were set to zero consistent with previous work (Chan et al., 2014; Malagurski et al., 2020). In addition, the removal of negative correlations is supposed to facilitate the interpretability of segregation metrics (Chan et al., 2014; Malagurski et al., 2020). Further, spurious interregional correlations were denoised by thresholding the connectivity matrices and preserving a proportion of 50% of the strongest weights. All connectomes were z-scored before subsequent analysis. All the other weights and all the weights on the main diagonal (self-connections) were set to zero as well. Detailed descriptions for HCHS connectome reconstructions were previously published (Petersen et al., 2022). For a detailed exploration of the impact of correlation matrix scenario without thresholds or with a threshold preserving 70 % for the strongest weights, please refer to Supplementary material.

### Measurement of structural and functional connectivity and functional segregation

Both the reconstructed structural and functional brain connectomes comprised nodes representing the same brain regions. Network edges in structural connectomes represented white matter (WM) microstructural integrity as the number of streamlines physically interconnecting network nodes, defined as structural connectivity. In functional connectomes, the edges represent synchronous neuronal activation defined as resting-state functional connectivity. Connectivity of structural and functional connectomes was calculated by taking the mean of the edges between all nodes in the matrix that were not excluded. Concerning the functional measurements, whole brain connectivity included all edges of the matrix, while global within connectivity comprised all edges within the seven resting state networks, and global between connectivity encompassed all edges between the seven resting state networks. The specific connectivity of the selected five resting state networks included all edges within the corresponding network.

We quantified functional network segregation throughout the whole cortex by using a metric formally defined as the difference between the mean within-network connectivity and mean between-network connectivity, divided by the mean within-network, noted in the following formula:


$$S\,e\,g\,r\,e\,g\,a\,t\,i\,o\,n=\frac{{\bar{Z}}_{w}-{\bar{Z}}_{b}}{{\bar{Z}}_{w}}$$

Z_*w*_ is the mean Fisher z-transformed r between nodes within all resting state networks and Z_*b*_ is the mean Fisher z-transformed r between nodes of all resting state to all nodes in other networks (Chan et al., 2014; Malagurski et al., 2020). Accordingly, system segregation for each resting state network was computed as the relative strength of functional connections within network in relation to connections between regions in the network and all other networks. Accordingly, this results in a segregation index for each network. From the segregation values of the individual networks, the global segregation for the entire brain was calculated by averaging the values of the functional segregation over all networks. Higher values of the segregation index reflect greater within- than between-network connectivity, and thus greater network segregation, while lower values represent a lower difference in within-to between- network connectivity and thus lower functional segregation.

**FIGURE 1:**
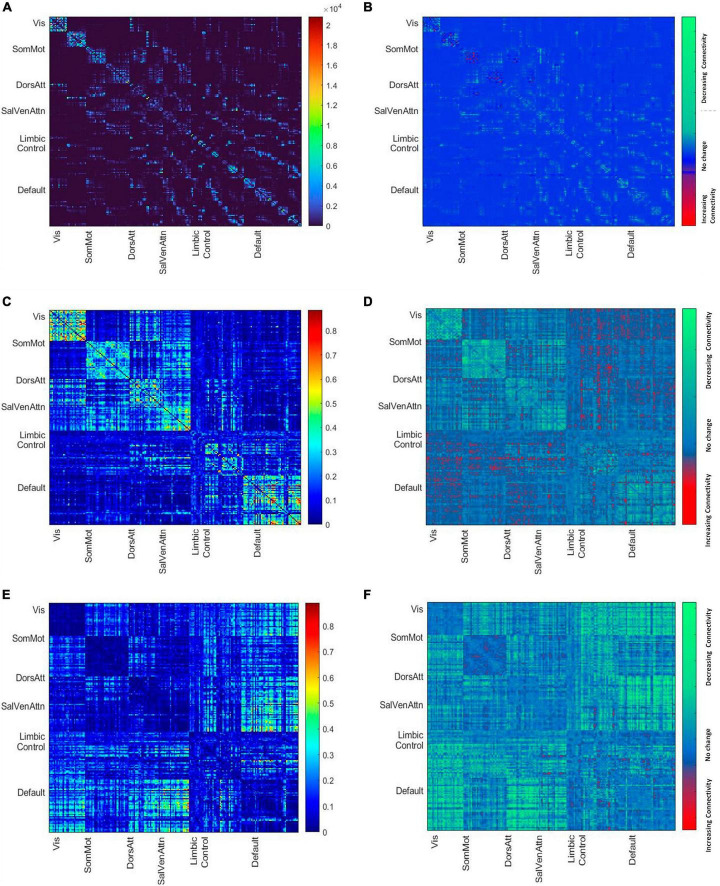
**(A)** Mean node-to-node structural matrices. Names along axes represent network labels of all seven resting state networks and the color bar shows the number of streamlines. **(B)** The difference map shows the decreasing and increasing number of streamlines between younger and older brains based on the subdivision of the cohort by median age. **(C)** Mean node-to-node correlation matrix of the whole cohort. The color bar shows the correlation strength. **(D)** The difference map shows the decreasing and increasing positive connectivity between younger and older brains based on the subdivision of the cohort by median age. **(E)** Mean node-to-node anti correlation matrix of the whole cohort. The color bar shows the anti-correlation strength. **(F)** The difference map shows the decreasing and increasing negative connectivity between younger and older brains based on the subdivision of the cohort by median age.

In addition, as a measurement of control, we constructed connectivity matrices with negative correlation values (see Figures 1E, F and in the Supplementary Table 1).

### Trail making test

The trail making test part B (TMT-B) is a measure of executive cognitive control (Reitan, 1958). The TMT-B requires the subject to connect 25 encircled numbers and letters in numerical and alphabetical order, alternating between the numbers and letters which are randomly distributed in space. The subject is also asked to connect the array of circles as quickly as possible without lifting the pencil. During the tests, the examiner corrects each error immediately (Terada et al., 2013). In trail making test, a shorter completion time is considered a gauge of better cognitive performance and is measured in seconds. In addition, a time limit of 300 s was set for the completion of the test. The results were then logarithmically transformed to ensure a normal distribution for the statistical analysis.

### Statistical analysis

All statistical analyses were conducted using R Version 4.0.2. In an initial step, we used univariable analyses to examine the influence of age on all imaging measurements and the cognitive TMT-B score. To assess the relationship between altered functional connectivity and functional segregation as the dependent variables and structural connectivity as the predictor, we first applied several multiple linear regressions, adjusted for age, sex and education. These linear models were calculated for global functional segregation and separately for the whole brain functional connectivity, the mean connectivity within all resting state networks and the mean connectivity between all resting state networks.

Subsequently mediation analyses using the Lavaan package (Rosseel, 2012) were conducted to further interrogate whether the association between age and the three functional connectivity measures or age and functional segregation is mediated by structural connectivity on global scale.

In the second part of the analysis, multiple regressions were used to investigate whether global functional connectivity, the mean connectivity within all resting state networks, the mean connectivity between all resting state networks, and functional segregation are directly associated with the TMT-B score adjusted for age, sex, and education.

Finally, we assessed the possible role of all three functional connectivity parameters and functional segregation as a mediator between age and executive cognitive function adjusted for, sex, education and structural connectivity. In all mediation analysis, we used non-parametric bootstrapping with 5,000 iterations to estimate direct and indirect associations between variables.

### Data availability statement

Anonymized data of the analysis not published within this article will be made available on reasonable request from any qualified investigator after evaluation of the request by the Steering Board of the HCHS.

## Results

### Sample characteristics

The demographics for the entire HCHS cohort and the median values of the cognitive test are shown in Table 1. The median age was 65 years (IQR = 14), 44% of the participants were female and the median years of education were 13 years (IQR 14). The mean structural and functional connectivity matrix of all participants is shown in Figure 1 along with difference maps Figures 1C, D showing the difference in structural and functional connectivity between younger and older participants of the cohort (see Figure 1).

**TABLE 1:** Sample characteristics and image analysis results–subjects used for MRI analysis of the Hamburg City Health Study.

Sample characteristics (*N* = 2445)	
Female sex [*n*, (%)]	985 (44%)
Age (years), median (IQR)	65 (14)
Education (years), median (IQR)	13 (4)
TMT B score (seconds), median (IQR)	81 (42)
MMSE	28 (20–30)
CDT	7 ± 1.1
Diastolic Blood Pressure	83 (13)

IQR, interquartile range; mm, millimeter; TMT, trail making test; MMSE, mini mental state examination; CDT, clock drawing test.

### Effects of age on structural and functional connectivity as well as on functional segregation and executive function

In an univariate analysis, age revealed a significant effect on all parameters as shown in the Table 2.

**TABLE 2:** Analysis results–for the univariate regression, representing unstandardized coefficient estimate (beta), SE, standardized estimate (standard beta), the *p*-value, and the *r*^2^ value.

All results univariate effect of age					
**Variables**	**Estimates**	**Std. estimate**	**Std. error**	***p*-value**	***r*** ^2^
**Direct univariate association of age with structural connectivity**					
Mean connectivity	−0.889	−0.42	0.04	*P* < 0.001	0.018
**Direct univariate association of age with functional connectivity and segregation**					
Mean connectivity	−0.0002	−0.13	0.00004	***P*** < **0.001**	0.016
Within network connectivity	−0.0014	−0.21	0.0001	***P*** < **0.001**	0.046
Between network connectivity	−0.00007	−0.03	0.00006	*P* = 0.224	0.0007
Mean segregation	− 0.003	−0.23	0.0002	***P*** < **0.001**	0.053
**Direct univariate association of age with the logarithmized** **TMTB Score**					
TMT B	1.7	037	0.0003	***P*** < **0.001**	0.14

The bold values represent significant results.

Of particular importance, since we used only the positive correlation values, we might not have measured age-related alterations due to negative or anti-correlation. Therefore, as a measure of control, we investigated the relationship between age and whole global functional connectivity, as well as separated into within and between network connectivity with negative matrix values. Supplementary Table 4 in the Supplementary reports the results (see in the Supplementary Table 1) and Figures 1E, F shows the mean functional connectivity matrices with negative values of all participants and the change in functional anticorrelation between younger and older participants of the cohort, revealing an overall decrease of anticorrelation between but not within resting state networks with increasing age (see Figures 1E, F).

### Direct influence of structural connectivity on functional connectivity and functional segregation, adjusted for age, sex and education

To analyze the direct association of structural connectivity on functional segregation and functional connectivity, we performed multiple linear regression analyses, adjusted for age sex and education. We iteratively tested the model with global mean functional connectivity, functional connectivity within all networks, functional connectivity between all networks and with global functional segregation.

While structural connectivity exhibited a direct association with functional segregation (*r*^2^ = 0.06, *r* = 0.04, *p* = 0.01), no such relationship was observed between structural connectivity and global mean functional connectivity (*r*^2^ = 0.02, *r* = −0.002, *p* = 0.95), global within network connectivity (*r*^2^= 0.05, *r* = 0.022, *p* = 0.26) or global between network connectivity (*r*^2^ = 0.0035, *r* = −0.03, *p* = 0.12) (see Table 3). To confirm the age-independence of this association, the interaction term between age structural connectivity in relation to functional segregation was examined in an additional model and was found to be non-significant (*p* = 0.47). Again, this analysis was also repeated with correlation matrices consisting exclusively of anti-correlations which showed no association of negative functional connectivity with structural connectivity (see in the Supplementary Table 1).

**TABLE 3:** Analysis results–for the multiple regression as well as the mediation analysis, representing unstandardized coefficient estimate (beta), SE, standardized estimate (standard beta) and the *p*-value.

Dependent variables	Estimates	Std. Estimate	Std. Error	*p*-value
**Direct association of structural connectivity with functional measurements on global scale**				
Global functional connectivity	−0.000001	−0.0015	0.00002	*p* = 0.95
Within network functional connectivity	0.00008	0.026	0.00007	*p* = 0.26
Between network functional connectivity	0.00005	−0.037	0.00003	*p* = 0.12
Global functional segregation	0.0002	0.06	0.00009	***p* = 0.0137**
**Mediation of structural connectivity between age and functional measurements on global scale**				
**Total association**				
Global functional connectivity	<−0.001	−0.123	<0.001	***P*** < **0.001**
Within network functional connectivity	<−0.001	−0.207	<0.001	***P*** < **0.001**
Between network functional connectivity	<−0.001	−0.024	<0.001	*p* = 0.25
Global functional segregation	−0.002	−0.022	<0.001	***P*** < **0.001**
**Direct association**				
Global functional connectivity	<−0.001	−0.123	<0.001	***P*** < **0.001**
Within network functional connectivity	−0.001	−0.196	<0.001	***P*** < **0.001**
Between network functional connectivity	<−0.001	−0.04	<0.001	*p* = 0.084
Global functional segregation	−0.002	−0.196	<0.001	***P*** < **0.001**
**Indirect association**				
Global functional connectivity	<0.001	0.001	<0.001	*P* = 0.949
Within network functional connectivity	<−0.001	−0.011	<0.001	*P* = 0.262
Between network functional connectivity	<0.001	0.016	<0.001	*P* = 0.099
Global functional segregation	<−0.001	−0.024	<0.001	***P* = 0.03**
**Direct association of functional measurements with the logarithmized** **TMTB score on global scale**				
Global functional connectivity	−0.6	−0.03	0.46	*P* = 0.19
Within network functional connectivity	−0.25	−0.04	0.14	*P* = 0.07
Between network functional connectivity	0.002	0.0001	0.33	*P* = 0.99
Global functional segregation	−0.24	−0.04	0.1	***P* = 0.03**
**Mediation of functional measurements between age and TMTB score**				
**Total association**				
Global functional connectivity	0.017	0.363	0.001	***P*** < **0.001**
Within network functional connectivity	0.017	0.363	0.001	***P*** < **0.001**
Between network functional connectivity	0.017	0.363	0.096	***P*** < **0.001**
Global functional segregation	0.017	0.364	0.001	***P*** < **0.001**
**Direct association**				
Global functional connectivity	0.016	0.360	0.001	***P*** < **0.001**
Within network functional connectivity	0.016	0.356	0.001	***P*** < **0.001**
Between network functional connectivity	0.017	0.363	0.096	***P*** < **0.001**
Global functional segregation	0.016	0.354	0.001	***P*** < **0.001**
**Indirect association**				
Global functional connectivity	<0.001	0.003	<0.001	*P* = 0.211
Within network functional connectivity	<0.001	0.007	<0.001	*P* = 0.1
Between network functional connectivity	<−0.001	<0.001	<0.001	*P* = 0.99
Global functional segregation	<0.001	0.1	<0.001	***P* = 0.032**

The bold values represent significant results.

### Structural connectivity as a mediator between age and functional connectivity and segregation, adjusted for sex and education

To assess whether structural connectivity acts as a mediator on the relation between age and functional connectivity or functional segregation we performed a mediation analysis. At the global level, age was the predictor of the outcome variable global functional segregation or of the three measures of global functional connectivity, respectively. Structural connectivity across the cortex acted as a possible mediator.

**FIGURE 2:**
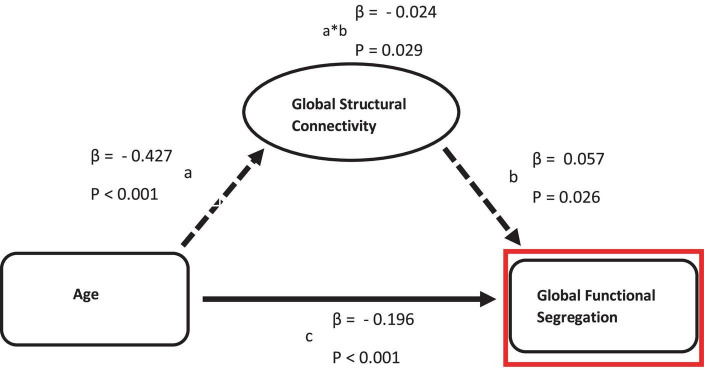
Mediation of the relation between age and functional measurements by structural connectivity on global scale. Path a: relationship between age and structural connectivity. Path b: relationship between structural connectivity and functional measurements. Path c: direct relationship between age and functional measurements.

Across the whole cortex, structural connectivity showed a significant but weak (β = −0.024) partial mediation relationship between age and functional segregation (see Figure 2). In contrast, no mediation relationship was observed between age and any of the three functional connectivity measures.

Furthermore, the network-specific structural connectivity was exploratively investigated as a potential mediator of the relationship between age and network-specific functional segregation or network-specific functional connectivity in five associative resting state networks (Default, Dorsal, Salience, Control, and Limbic network). Solely the network specific structural connectivity of the default mode network revealed a partial mediation effect (see in the Supplementary Figure 1A).

### Direct influence of functional connectivity and functional segregation on executive cognitive function, adjusted for age, sex, education and structural connectivity

In the second part of the analysis, we conducted multiple linear regressions to investigate a possible direct association of all three global functional connectivity measures or global functional segregation on executive cognitive function in the form of the TMT B score. These models were controlled for age, sex and education.

Global functional segregation showed a direct influence on the TMT B score (*r*^2^ = 0.2, *r* = −0.044, *p* = 0.03). At the same time, neither global mean functional connectivity (*r*^2^ = 0.2, *r* = −0.009, *p* = 0.19), global within network connectivity (*r*^2^ = 0.2, *r* = −0.022, *p* = 0.7), nor global between network connectivity (*r*^2^ = 0.2, *r* = 0.016, *p* = 0.99) exhibited an association with the cognitive score (see Table 3).

### Functional connectivity and functional segregation as a mediator between age and executive cognitive function, adjusted for sex, education and structural connectivity

Finally, to investigate whether one of the functional parameters mediates the relationship of age with the TMT B score, we conducted a second mediation analysis.

Age was the predictor for the outcome variable TMT B-score, while on global scale, functional segregation and all three measures of functional connectivity were possible mediators.

**FIGURE 3:**
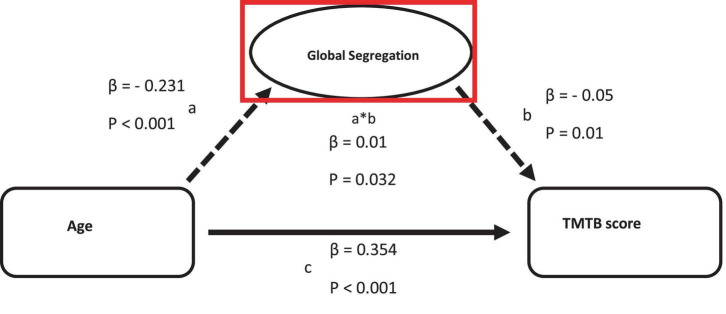
Mediation of the relation between age and TMTB score by functional measurements on global scale. Path a: relationship between age and the logarithmized TMTB score. Path b: relationship between functional measurements and TMTB score. Path c: direct relationship between age and TMTB score.

Decreasing global functional segregation was the only parameter exhibiting a partial mediation relationship between age and executive cognitive function (β = −0.012) (see Figure 3).

Again, network-specific functional connectivity and network-specific functional segregation were exploratively investigated in the five resting state networks as potential mediators between age and TMTB. Both the network-specific functional connectivity and the network-specific segregation of the default mode network showed a partial mediation effect between age and the TMTB score. The significant results of this analysis are shown in the (see in the Supplementary Figures 1B, C).

### Alternative network thresholding

Notably, the results remained consistent across different threshold settings (no threshold, threshold of 0.3), demonstrating the robustness of our analyses (see in the Supplementary Tables 2, 3). This approach is consistent with previously published work by Chan et al. (2014) and Madden et al. (2020), which are referenced in the manuscript.

## Discussion

Gaining a better understanding of how age-related effects of structural integrity affect functional activity patterns of the brain is crucial to expand our scientific knowledge of the ageing process. Therefore, in the present study, we analyzed in a first step the association of age-related effects in structural connectivity with functional connectivity and with dedifferentiation or loss of functional specialization of brain networks. We found a significant age-independent association between decreasing structural connectivity and decreasing functional segregation across the brain, as well as a mediation relationship of structural connectivity between age and global functional segregation. On the contrary, we did not observe any influence of structural connectivity on functional connectivity. In a second step, we examined the relationship of functional connectivity, as well as functional segregation with executive cognitive function measured by the TMT-B score. Global functional segregation showed both a direct association with executive cognitive function and a partial mediation relationship between increasing age and the cognitive score. No such association was observed between functional connectivity and executive cognitive function at the global level.

The “normal” ageing of the brain is associated with a variety of physical, biological, and chemical changes throughout an individual’s life (Huang et al., 2015), leading to alteration in structural, functional, and cognitive abilities (Schulz et al., 2022). Consistent with results from other studies (Madden et al., 2009; Onoda et al., 2012; Sexton et al., 2014; Farras-Permanyer et al., 2019), we observed decline of structural and functional connectivity on global scale. In addition, our findings complement previous reports, which found a global decline in segregation, particular in the associative networks (Chan et al., 2014; Wig, 2017; Pedersen et al., 2021). A more controversial observation is the development of inter-network connectivity which revealed no significant alteration with age and thus contrasts with other studies that observed increasing connectivity between resting state networks (Betzel et al., 2014; Chan et al., 2014). However, by looking at positive and negative correlation separately in our study, we were able to detect a significant decrease in negative correlations between networks. This may suggest that the increase in connectivity between networks in other studies is driven by a decrease in anti-correlations rather than an increase in positive correlations. In addition, due to the range of age in this cohort of 25 years and the cross-sectional character of the study, only a section of the functional alterations between the networks with increasing age can be represented and limits the possibility of recognizing more dynamic developments.

Regarding changes in functional connectivity measures, we largely reproduce findings of the literature on age-related loss of coordinated neural activity between brain regions. This temporal-spatial organization of neuronal activity in the brain is thought to be supported and constrained by the anatomy of axonal projections that form structural connections between both the neighboring and distant brain regions (van den Heuvel and Sporns, 2019; Suárez et al., 2020). However, even though the “disconnection hypothesis” suggests that impaired integrity of these structural pathways leads to a deficit in functional coordination, it must be recognized that functional connectivity does not simply coincide with structural connectivity and that new functional biomarkers must also be considered. With the aim to link the organization of physical connections to patterns of functional interactions, we observed a direct association of decreasing structural connectivity with decreasing functional segregation between networks. The fact that, conversely, no association was found between structural and functional connectivity at the same time suggests that functional segregation is a more sensitive biomarker of functional change in older age which is in line with our hypothesis. This hypothesis is based on the suggestion of Madden et al. (2020) that the decrease in functional network segregation may be the result of reduced structural efficiency, with functional connections increasingly relying on indirect pathways.

In this regard, it is reasonable to assume that direct functional connectivity within resting state networks is eroded by age-related disruptions in the structural wiring of the brain, and that functional integrity is more and more maintained through indirect pathways. However, these indirect pathways include regions outside of each network, which subsequently leads to less functional differentiation between the macroscale resting state networks. While the anatomical alignment of structural and functional connectivity measures seems to be limited to specific networks and pathways according to past findings (Fjell et al., 2017), our study indicates a reliable global pattern between decreasing structural integrity and decreasing functional segregation. This impression is reinforced by additional results of our study, revealing a partial mediator role of structural connectivity between age and functional segregation, whereas structural connectivity did not appear as a mediator between age and functional connectivity. The direct association of structural connectivity and functional segregation, as well as the role of structural connectivity as a mediator between age and segregation, confirm and extend the findings of Pedersen et al. (2021) who reported in a longitudinal analysis an association between structural integrity and functional segregation (Pedersen et al., 2021), as well as the analysis from Kong et al. (2020) which was linking cerebral vascular elasticity, white matter lesions and cortical thickness to functional segregation.

The age-related shifts in the functional activity of the macroscopic functional brain networks shown here are not without cognitive consequences. Although neurocognitive changes involve multiple mechanisms, it can be generalized from several task-based imaging studies that brain regions that show specialized responses to specific cognitive processes in young adults tend to be less specialized in older adults and respond similarly to a wider range of different cognitive conditions, representing an age-related de-differentiated functional response to cognitive challenges (Park et al., 2001, 2004; Cabeza, 2002; Li and Sikström, 2002). Our results reveal a direct relationship between decreasing global segregation and more time to complete the TMT-B test and additionally suggest that the relationship between age and TMT-B score is mediated by functional segregation while neither of these findings could be shown with functional connectivity. These results are consistent with the idea that reduced specificity of cognitive measures may be a consequence of de-differentiation in brain networks (Pedersen et al., 2021) and are underpinned by past analyses suggesting that functional dedifferentiation is associated with slower perceptual comparison time in older people as well as the inability to avoid the propagation of internal or external noise that could potentially impair task-relevant functions (Stevens et al., 2012; JSTOR, 2022), resulting in older people needing more time to identify similarities between visual stimuli (Park et al., 2004).

Taken together, this analysis suggests that the segregation metric is a more sensitive biomarker of cognitive ageing than functional connectivity on global scale and provides a unique and more complementary network-based explanation for the widely reported cognitive decline with age.

However, the association of functional segregation with both structural connectivity and executive cognition observed was rather weak. Our results corroborate previous studies indicating a limited correspondence between functional segregation and structural connectivity in ageing (Madden et al., 2020; Pedersen et al., 2021), and are related to reports suggesting that a direct structural connection between two brain regions does not explain more than 50% of the variance in functional connectivity patterns between these regions (Suárez et al., 2020). Furthermore, the structural and functional properties of the brain studied here are based on nodes that are identical in the representation of networks. However, the brain regions these nodes represent differ in local features such as gene transcription, cytoarchitecture, receptor profiles and temporal dynamics that in turn influence how neuronal populations transmit and integrate signaling (Suárez et al., 2020), which can lead to regional differences in macroscale functional patterns independent of the structural basis. Therefore, the macroscopic representation of functional networks composed of relatively large brain regions is unlikely to contain the biological details necessary for a more precise characterization of the relationship between structural integrity and functional change. Further research on functional segregation is needed. The discordance between structure and function is diverging at the mesoscopic scale and vary in parallel with cytoarchitectonic and representational hierarchies (Suárez et al., 2020). As a result, the exact nature of interaction between structure and function is probably obscured at the macroscopic level by the methods commonly used to define structural and functional network properties. In addition, conventional static and dyadic representation of functional activity employed here as in most studies may not adequately capture the dynamic character of functional interactions between neuronal regions. Future analyses that take regional heterogeneity into account by enriching network reconstructions with microscale attributes and working with metrics based on dynamic functional connectivity will provide a sharper picture of declining structural integrity and increasing functional blurring between functional networks.

## Data availability statement

The raw data supporting the conclusions of this article will be made available by the authors, without undue reservation.

## Ethics statement

The studies involving humans were approved by the Landesärztekammer Hamburg (State of Hamburg Chamber of Physicians, PV5131). The studies were conducted in accordance with the local legislation and institutional requirements. The participants provided their written informed consent to participate in this study.

## Author contributions

MS: Conceptualization, Formal Analysis, Investigation, Methodology, Software, Visualization, Writing – original draft. MP: Data curation, Methodology, Software, Writing – review and editing. BC: Writing – review and editing. GT: Funding acquisition, Supervision, Writing – review and editing.
