# Supplementary figures and images for: Association of structural connectivity with functional brain network segregation in a middle-aged to elderly population
Source: Front Aging Neurosci. 2024 Feb 1;16:1291162. doi: 10.3389/fnagi.2024.1291162 (PMC10870644; doi:10.3389/fnagi.2024.1291162)

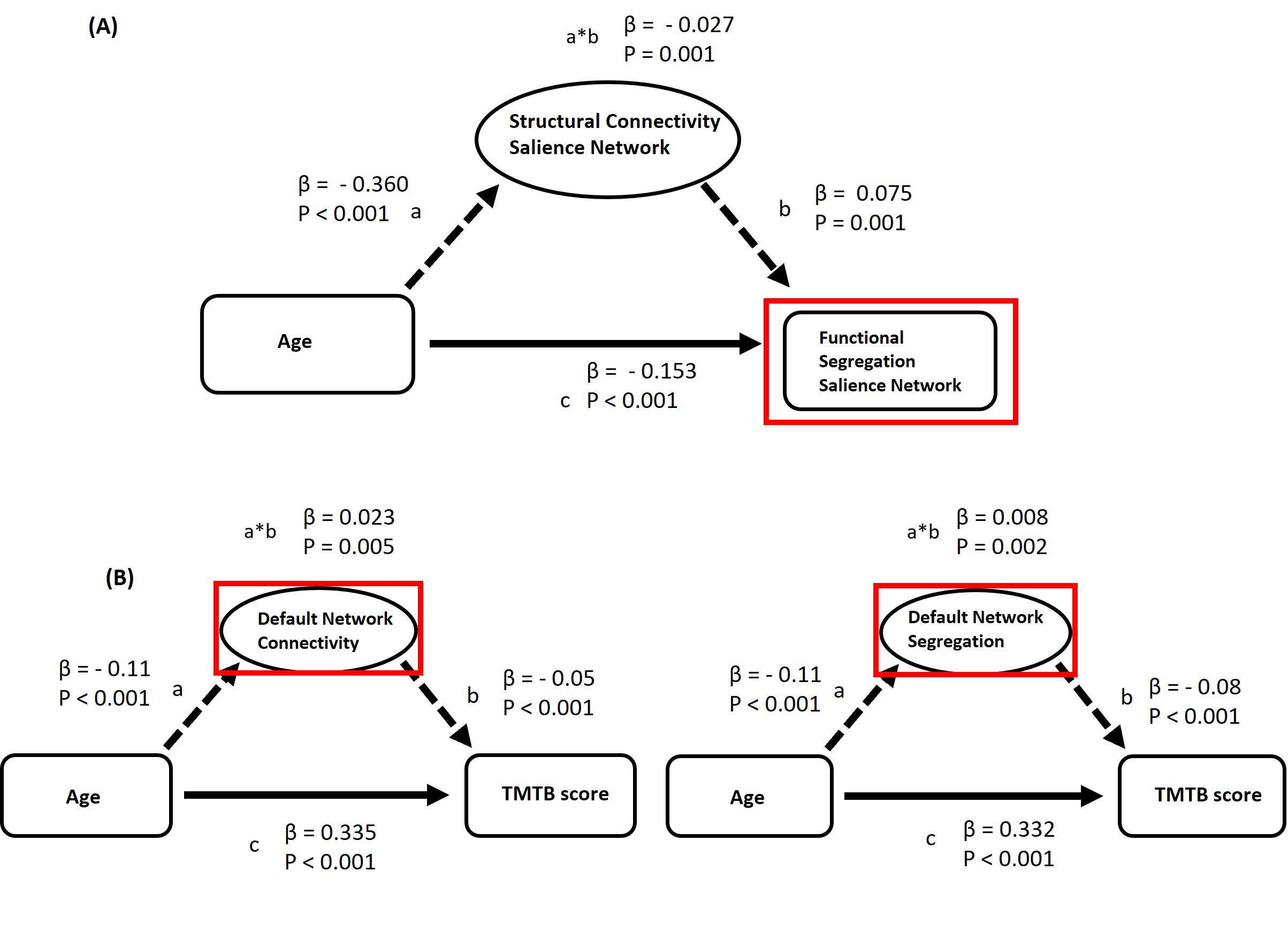

Supplement: Supplementary file 1 [file Image_1.JPEG]
