# Supplementary material for: Association of structural connectivity with functional brain network segregation in a middle-aged to elderly population
Source: Front Aging Neurosci. 2024 Feb 1;16:1291162. doi: 10.3389/fnagi.2024.1291162 (PMC10870644; doi:10.3389/fnagi.2024.1291162)
Supplement: Supplementary file 2 [file Table_1.docx]

| **All Results Anti-Correlation** | | | | |
| --- | --- | --- | --- | --- |
| **Variables** | **Estimates** | **Std. Estimate** | **Std. Error** | **p-Value** |
| **Direct univariate association of age with negative functional connectivity on global scale** | | | | |
| Mean connectivity | - 0.0002 | - 0.16 | 0.00004 | **P < 0.001** |
| Within network connectivity | 0.00008 | 0.03 | 0.00005 | P = 0.18 |
| Between network connectivity | - 0.0003 | - 0.13 | 0.00005 | **P < 0.001** |
| **Direct association of structural connectivity with negative functional connectivity on global scale** | | | | |
| Mean connectivity | 0.00003 | 0.03 | 0.00004 | P = 0.16 |
| Within network connectivity | - 0.00005 | - 0.03 | 0.00004 | P = 0.18 |
| Between network connectivity | 0.00002 | 0.016 | 0.00003 | P = 0.5 |
| **Mediation of structural connectivity between age and negative functional connectivity on global scale** | | | | |
| **Total Association** |  |  |  |  |
| Mean connectivity | < - 0.001 | - 0.155 | < - 0.001 | **P < 0.001** |
| Within network connectivity | < - 0.001 | 0.024 | < - 0.001 | P = 0.272 |
| Between network connectivity | < - 0.001 | - 0.123 | < - 0.001 | **P < 0.001** |
| **Direct Association** |  |  |  |  |
| Mean connectivity | < - 0.001 | - 0.141 | < - 0.001 | P = 0.141 |
| Within network connectivity | < - 0.001 | 0.024 | < - 0.001 | P = 0.272 |
| Between network connectivity | < - 0.001 | - 0.116 | < - 0.001 | **P < 0.001** |
| **Indirect Association** |  |  |  |  |
| Mean connectivity | < - 0.001 | - 0.014 | < - 0.001 | **P < 0.001** |
| Within network connectivity | < - 0.001 | 0.024 | < - 0.001 | P = 0.272 |
| Between network connectivity | < - 0.001 | - 0.007 | < - 0.001 | P = 0.161 |
| **Direct association of negative functional connectivity with the logarithmized TMTB score on global scale** | | | | |
| Mean connectivity | - 76.45 | - 0.03 | 55.1 | P = 0.17 |
| Within network connectivity | 24.86 | 0.014 | 33.56 | P = 0.46 |
| Between network connectivity | - 26.23 | - 0.014 | 34.57 | P = 0.45 |
| **Mediation of negative functional connectivity between age and the logarithmized TMTB score** | | | | |
| **Total Association** |  |  |  |  |
| Mean connectivity | 1.464 | 0.321 | 0.096 | **P < 0.001** |
| Within network connectivity | 1.463 | 0.321 | 0.099 | **P < 0.001** |
| Between network connectivity | 1.463 | 0.321 | 0.097 | **P < 0.001** |
| **Direct Association** |  |  |  |  |
| Mean connectivity | 1.445 | 0.317 | 0.097 | **P < 0.001** |
| Within network connectivity | 1.461 | 0.320 | 0.099 | **P < 0.001** |
| Between network connectivity | 1.456 | 0.319 | 0.097 | **P < 0.001** |
| **Indirect Association** |  |  |  |  |
| Mean connectivity | 0.019 | 0.004 | 0.015 | P = 0.198 |
| Within network connectivity | 0.002 | < - 0.001 | 0.003 | P = 0.54 |
| Between network connectivity | 0.008 | 0.002 | 0.011 | P = 0.47 |

**Table 1.** Analysis results for anticorrelation - for the multiple regression as well as the mediation analysis, representing unstandardized coefficient estimate (beta), SE, standardized estimate (standard beta), the *p*-value, and the *r*^2^ value.
