# Supplementary material for: Association of structural connectivity with functional brain network segregation in a middle-aged to elderly population
Source: Front Aging Neurosci. 2024 Feb 1;16:1291162. doi: 10.3389/fnagi.2024.1291162 (PMC10870644; doi:10.3389/fnagi.2024.1291162)
Supplement: Supplementary file 3 [file Table_2.docx]

| **All Results Zero Threshold** | | | | |
| --- | --- | --- | --- | --- |
| **Variables** | **Estimates** | **Std. Estimate** | **Std. Error** | **p-Value** |
| **Direct univariate association of age with functional measurements on global scale** | | | | |
| Mean connectivity | - 0.0002 | - 0.13 | 0.00005 | **P < 0.001** |
| Within network connectivity | - 0.001 | - 0.22 | 0.0001 | **P < 0.001** |
| Between network connectivity | - 0.0001 | - 0.47 | 0.00005 | **P = 0.03** |
| Mean Segregation | - 0.002 | - 0.22 | 0.0002 | **P < 0.001** |
| **Direct association of structural connectivity with functional measurements on global scale** | | | | |
| Mean connectivity | 0.000002 | 0.002 | 0.00002 | P = 0.94 |
| Within network connectivity | 0.00009 | 0.03 | 0.00007 | P = 0.2 |
| Between network connectivity | - 0.00003 | - 0.028 | 0.00003 | P = 0.2 |
| Mean Segregation | 0.0002 | - 0.054 | 0.00009 | **P = 0.02** |
| **Mediation of structural connectivity between age and negative functional connectivity on global scale** | | | | |
| **Total Effect** |  |  |  |  |
| Mean connectivity | < - 0.001 | - 0.122 | < 0.001 | **P < 0.001** |
| Within network connectivity | < - 0.001 | - 0.212 | < 0.001 | **P < 0.001** |
| Between network connectivity | < - 0.001 | - 0.045 | < 0.001 | **P = 0.03** |
| Mean Segregation | 0.002 | 0.21 | < 0.001 | **P < 0.001** |
| **Direct Effect** |  |  |  |  |
| Mean connectivity | < - 0.001 | - 0.123 | < 0.001 | **P < 0.001** |
| Within network connectivity | 0.001 | - 0.199 | < 0.001 | **P < 0.001** |
| Between network connectivity | < - 0.001 | - 0.057 | < 0.001 | **P = 0.01** |
| Mean Segregation | 0.002 | - 0.19 | < 0.001 | **P < 0.001** |
| **Indirect Effect** |  |  |  |  |
| Mean connectivity | < 0.001 | 0.001 | < 0.001 | P = 0.942 |
| Within network connectivity | < - 0.001 | - 0.013 | < 0.001 | P = 0.221 |
| Between network connectivity | < 0.001 | 0.012 | < 0.001 | P = 0.206 |
| Mean Segregation | < - 0.001 | 0.02 | < 0.001 | **P = 0.04** |
| **Direct association of negative functional connectivity with the logarithmized TMTB score on global scale** | | | | |
| Mean connectivity | - 0.42 | - 0.02 | 0.48 | P = 0.4 |
| Within network connectivity | - 0.2 | - 0.03 | 0.14 | P = 0.15 |
| Between network connectivity | 0.2 | - 0.01 | 0.35 | P = 0.6 |
| Mean Segregation | - 0.24 | - 0.04 | 0.11 | **P = 0.03** |
| **Mediation of negative functional connectivity between age and the logarithmized TMTB score** | | | | |
| **Total Effect** |  |  |  |  |
| Mean connectivity | 0.016 | 0.355 | 0.001 | **P < 0.001** |
| Within network connectivity | 0.016 | 0.356 | 0.001 | **P < 0.001** |
| Between network connectivity | 0.016 | 0.356 | 0.001 | **P < 0.001** |
| Mean Segregation | 0.016 | 0.356 | 0.001 | **P < 0.001** |
| **Direct Effect** |  |  |  |  |
| Mean connectivity | 0.016 | 0.354 | 0.001 | **P < 0.001** |
| Within network connectivity | 0.016 | 0.350 | 0.001 | **P < 0.001** |
| Between network connectivity | 0.016 | 0.356 | 0.001 | **P < 0.001** |
| Mean Segregation | 0.016 | 0.347 | 0.001 | **P < 0.001** |
| **Indirect Effect** |  |  |  |  |
| Mean connectivity | < 0.001 | 0.002 | < 0.001 | P = 0.52 |
| Within network connectivity | < 0.001 | 0.006 | < 0.001 | P = 0.2 |
| Between network connectivity | <- 0.001 | < - 0.001 | < 0.001 | P = 0.64 |
| Mean Segregation | < 0.001 | 0.009 | < 0.001 | **P = 0.03** |

**Table 2.**  Analysis results - for the network specific mediation analysis, representing unstandardized coefficient estimate (beta), SE, standardized estimate (standard beta) and the *p*-value.
