# Supplementary material for: Association of structural connectivity with functional brain network segregation in a middle-aged to elderly population
Source: Front Aging Neurosci. 2024 Feb 1;16:1291162. doi: 10.3389/fnagi.2024.1291162 (PMC10870644; doi:10.3389/fnagi.2024.1291162)
Supplement: Supplementary file 5 [file Table_4.docx]

| **All Results 50 % Threshold + cardiovascular risk factors (disastolic blood pressure)** | | | | |
| --- | --- | --- | --- | --- |
| **Variables** | **Estimates** | **Std. Estimate** | **Std. Error** | **p-Value** |
| **Direct association of structural connectivity with functional measurements on global scale** | | | | |
| Mean connectivity | 0.000008 | 0.009 | 0.00002 | P = 0.72 |
| Within network connectivity | 0.00009 | 0.04 | 0.00007 | P = 0.13 |
| Between network connectivity | - 0.00004 | - 0.03 | 0.00003 | P = 0.19 |
| Mean Segregation | 0.0002 | 0.06 | 0.00009 | P = 0.01 |
| **Mediation of structural connectivity between age and negative functional connectivity on global scale** | | | | |
| **Total Effect** |  |  |  |  |
| Mean connectivity | < - 0.001 | - 0.112 | < 0.001 | P < 0.001 |
| Within network connectivity | - 0.001 | - 0.2 | < 0.001 | P < 0.001 |
| Between network connectivity | < - 0.001 | - 0.045 | < 0.001 | P = 0.571 |
| Mean Segregation | - 0.002 | - 0.224 | < 0.001 | P < 0.001 |
| **Direct Effect** |  |  |  |  |
| Mean connectivity | < - 0.001 | - 0.108 | < 0.001 | P < 0.001 |
| Within network connectivity | - 0.001 | - 0.185 | < 0.001 | P < 0.001 |
| Between network connectivity | < - 0.001 | - 0.026 | < 0.001 | P = 0.261 |
| Mean Segregation | - 0.002 | - 0.199 | < 0.001 | P < 0.001 |
| **Indirect Effect** |  |  |  |  |
| Mean connectivity | < - 0.001 | - 0.004 | < 0.001 | P = 0.72 |
| Within network connectivity | < - 0.001 | - 0.016 | < 0.001 | P = 0.213 |
| Between network connectivity | < 0.001 | 0.014 | < 0.001 | P = 0.18 |
| Mean Segregation | < - 0.001 | - 0.025 | < 0.001 | p = 0.031 |
| **Direct association of negative functional connectivity with the logarithmized TMTB score on global scale** | | | | |
| Mean connectivity | - 0.48 | - 0.02 | 0.46 | P = 0.3 |
| Within network connectivity | - 0.19 | - 0.03 | 0.13 | P = 0.17 |
| Between network connectivity | 0.008 | 0.004 | 0.32 | P = 0.9 |
| Mean Segregation | - 0.21 | - 0.04 | 0.11 | P = 0.06 |
| **Mediation of negative functional connectivity between age and the logarithmized TMTB score** | | | | |
| **Total Effect** |  |  |  |  |
| Mean connectivity | 0.016 | 0.365 | 0.001 | P < 0.001 |
| Within network connectivity | 0.017 | 0.368 | 0.001 | P < 0.001 |
| Between network connectivity | 0.017 | 0.367 | 0.001 | P < 0.001 |
| Mean Segregation | 0.016 | 0.368 | 0.001 | P < 0.001 |
| **Direct Effect** |  |  |  |  |
| Mean connectivity | 0.016 | 0.367 | 0.001 | P < 0.001 |
| Within network connectivity | 0.017 | 0.362 | 0.001 | P < 0.001 |
| Between network connectivity | 0.017 | 0.367 | 0.001 | P < 0.001 |
| Mean Segregation | 0.016 | 0.36 | 0.001 | P < 0.001 |
| **Indirect Effect** |  |  |  |  |
| Mean connectivity | < 0.001 | 0.002 | < 0.001 | P = 0.312 |
| Within network connectivity | < 0.001 | 0.006 | < 0.001 | P = 0.18 |
| Between network connectivity | < - 0.001 | < - 0.001 | < 0.001 | P = 0.986 |
| Mean Segregation | < - 0.001 | 0.009 | < 0.001 | P = 0.05 |

**Table 4.**  Analysis results - for the network specific mediation analysis, representing unstandardized coefficient estimate (beta), SE, standardized estimate (standard beta) and the *p*-value.

| **All Results 50 % Threshold + cardiovascular riskfactors** | | | | |
| --- | --- | --- | --- | --- |
| **Variables** | **Estimates** | **Std. Estimate** | **Std. Error** | **p-Value** |
| **Direct association of structural connectivity with functional measurements on global scale** | | | | |
| Mean connectivity | 0.000008 | 0.009 | 0.00002 | P = 0.72 |
| Within network connectivity | 0.00009 | 0.04 | 0.00007 | P = 0.13 |
| Between network connectivity | - 0.00004 | - 0.03 | 0.00003 | P = 0.19 |
| Mean Segregation | 0.0002 | 0.06 | 0.00009 | P = 0.01 |
| **Mediation of structural connectivity between age and negative functional connectivity on global scale** | | | | |
| **Total Effect** |  |  |  |  |
| Mean connectivity | < - 0.001 | - 0.112 | < 0.001 | P < 0.001 |
| Within network connectivity | - 0.001 | - 0.2 | < 0.001 | P < 0.001 |
| Between network connectivity | < - 0.001 | - 0.045 | < 0.001 | P = 0.571 |
| Mean Segregation | - 0.002 | - 0.224 | < 0.001 | P < 0.001 |
| **Direct Effect** |  |  |  |  |
| Mean connectivity | < - 0.001 | - 0.108 | < 0.001 | P < 0.001 |
| Within network connectivity | - 0.001 | - 0.185 | < 0.001 | P < 0.001 |
| Between network connectivity | < - 0.001 | - 0.026 | < 0.001 | P = 0.261 |
| Mean Segregation | - 0.002 | - 0.199 | < 0.001 | P < 0.001 |
| **Indirect Effect** |  |  |  |  |
| Mean connectivity | < - 0.001 | - 0.004 | < 0.001 | P = 0.72 |
| Within network connectivity | < - 0.001 | - 0.016 | < 0.001 | P = 0.213 |
| Between network connectivity | < 0.001 | 0.014 | < 0.001 | P = 0.18 |
| Mean Segregation | < - 0.001 | - 0.025 | < 0.001 | p = 0.031 |
| **Direct association of negative functional connectivity with TMTB score on global scale** | | | | |
| Mean connectivity | - 0.48 | - 0.02 | 0.46 | P = 0.3 |
| Within network connectivity | - 0.19 | - 0.03 | 0.13 | P = 0.17 |
| Between network connectivity | 0.008 | 0.004 | 0.32 | P = 0.9 |
| Mean Segregation | - 0.21 | - 0.04 | 0.11 | P = 0.06 |
| **Mediation of negative functional connectivity between age and TMTB score** | | | | |
| **Total Effect** |  |  |  |  |
| Mean connectivity | 0.016 | 0.365 | 0.001 | P < 0.001 |
| Within network connectivity | 0.017 | 0.368 | 0.001 | P < 0.001 |
| Between network connectivity | 0.017 | 0.367 | 0.001 | P < 0.001 |
| Mean Segregation | 0.016 | 0.368 | 0.001 | P < 0.001 |
| **Direct Effect** |  |  |  |  |
| Mean connectivity | 0.016 | 0.367 | 0.001 | P < 0.001 |
| Within network connectivity | 0.017 | 0.362 | 0.001 | P < 0.001 |
| Between network connectivity | 0.017 | 0.367 | 0.001 | P < 0.001 |
| Mean Segregation | 0.016 | 0.36 | 0.001 | P < 0.001 |
| **Indirect Effect** |  |  |  |  |
| Mean connectivity | < 0.001 | 0.002 | < 0.001 | P = 0.312 |
| Within network connectivity | < 0.001 | 0.006 | < 0.001 | P = 0.18 |
| Between network connectivity | < - 0.001 | < - 0.001 | < 0.001 | P = 0.986 |
| Mean Segregation | < - 0.001 | 0.009 | < 0.001 | P = 0.05 |
